# Isolation of clade 2.3.4.4b A(H5N8), a highly pathogenic avian influenza virus, from a worker during an outbreak on a poultry farm, Russia, December 2020

**DOI:** 10.2807/1560-7917.ES.2021.26.24.2100439

**Published:** 2021-06-17

**Authors:** Olga G Pyankova, Ivan M Susloparov, Anastasia A Moiseeva, Natalia P Kolosova, Galina S Onkhonova, Aleksey V Danilenko, Elena V Vakalova, Gennady L Shendo, Natalia N Nekeshina, Lyudmila N Noskova, Julia V Demina, Natalia V Frolova, Elena V Gavrilova, Rinat A Maksyutov, Aleksandr B Ryzhikov

**Affiliations:** 1 State Research Centre of Virology and Biotechnology "Vector" Rospotrebnadzor, Koltsovo, Novosibirsk Region, Russia; 2 Hygienic and Epidemiological Centre of Astrakhan Region, Federal Service for Surveillance on Consumer Rights Protection and Human Wellbeing (Rospotrebnadzor) Astrakhan, Russia; 3 Astrakhan Regional office of Federal Service for Surveillance on Consumer Rights Protection and Human Wellbeing (Rospotrebnadzor), Astrakhan, Russia; 4 Federal Service for Surveillance on Consumer Rights Protection and Human Wellbeing (Rospotrebnadzor), Moscow, Russia

**Keywords:** avian influenza virus, HPAIV, H5N8, human influenza, surveillance, Clade 2.3.4.4, phylogenetic

## Abstract

This study presents the isolation of influenza A(H5N8) virus clade 2.3.4.4b from a poultry worker during an outbreak of highly pathogenic avian influenza A(H5N8) among chickens at a poultry farm in Astrakhan, Russia in December 2020. Nasopharyngeal swabs collected from seven poultry workers were positive for influenza A(H5N8), as confirmed by RT-PCR and sequencing. The influenza A(H5N8) virus was isolated from one of the human specimens and characterised. Sporadic human influenza A(H5)2.3.4.4. infections represent a possible concern for public health.

In December 2020, the RNA of avian influenza A(H5N8) virus was detected in nasopharyngeal swabs taken from seven poultry workers during an outbreak at a large poultry farm in the Astrakhan region on the Volga River in southern Russia. An influenza A(H5N8) virus isolate was obtained from one human clinical specimen.

## Epidemiological data

### Outbreak among poultry

The outbreak of highly pathogenic avian influenza (HPAI) occurred at the *Vladimirovskaya* poultry farm located in Astrakhan region, Russia. The total flock size at the poultry farm was 924,612 chickens of all ages. On 3 December 2020, the first 750 chickens died and additional mortality was later observed at the site. All birds had the same symptoms (diarrhoea, conjunctivitis, haemorrhagic lesions of the respiratory and digestive tract). The Astrakhan Veterinary Laboratory preliminarily detected influenza A(H5) viral RNA in poultry tissue samples (lung, trachea, intestine, spleen) from a subset of 18 dead birds on 7 December 2020. On the same day, upon receiving notification about the situation, the Russian Federal Service for Surveillance on Consumer Rights Protection and Human Wellbeing (Rospotrebnadzor) immediately ordered the suspension of production on the affected farm and a recall of live birds and poultry products produced on this farm from markets throughout the country. Therefore, by 11 December 2020, 101,000 poultry had died on the farm, and the remaining poultry was slaughtered. Influenza A(H5)-positive specimens from 10 chickens from various representative locations on the farm were selected for further analysis.

### Investigation of farm workers

Fifty-six farm workers had contact with dead birds or were present at the site since the start of the outbreak. On 12 December 2020, 56 serum samples and 37 nasopharyngeal swabs from these 56 poultry workers were collected simultaneously by the Akhtubinsk District Hospital (for 37 individuals, both serum and swabs were collected). Medical follow-up was established for 150 persons (the poultry farm workers and their family members), and prophylaxis with antiviral drugs was initiated for all 150. According to the district hospital, all of them remained asymptomatic during the 21-day follow-up period.

On 17 December 2020, 10 samples from 10 chickens and 56 serum samples and 37 nasopharyngeal swabs from poultry farm workers were transferred to the State Research Centre of Virology and Biotechnology ‘Vector’ for virological analysis. Serum was re-sampled 14 and 44 days after 12 December 2020 from seven poultry workers in whose swabs influenza A RNA had been detected. The seven persons were aged between 29 and 60 years, five were female and two were male.

Environmental specimens were not collected because this is not currently foreseen in the Veterinary Laboratory’s epizootic protocols.

## Molecular analysis

The presence of influenza A(H5) viral RNA was detected by RT-PCR (Interlabservice, Moscow, Russia) in all 10 analysed avian samples. Seven human nasopharyngeal swabs were positive for influenza A, five of which were positive for influenza A(H5) in RT-PCR analysis. All seven human samples were subtyped as influenza A(H5N8) by partial sequencing of the haemagglutinin (HA) and neuraminidase (NA) gene amplicons obtained using nested PCR (Supplementary Table S1).

Avian influenza viruses were isolated from 10 avian samples after one passage in an embryonated chicken egg (ECE). No viruses were isolated from human samples after three passages in ECE. Only one influenza virus, A/Astrakhan/3212/2020(H5N8), was isolated from our human samples when propagated in Madin-Darby Canine Kidney (MDCK) cells. To exclude contamination, virus isolation was performed according to the World Health Organization (WHO) manual under strict adherence to all guidelines, in particular processing of human and animal specimens in separate laboratories and inclusion of all necessary negative controls [1].

Serum samples from poultry workers were tested in a focus reduction neutralisation assay (FRNA) in MDCK cells with result detection by nucleoprotein immunostaining in the cell monolayer 24 h after infection [1], by haemagglutination inhibition assay (HIA) with horse red blood cells [1], and by biolayer interferometry (BLI) using the Octet RED96e system (Pall ForteBio, Fremont, United States) described in the Supplement. All serological tests were performed with the influenza strain A/Astrakhan/3212/2020.

The analysis of paired serum samples in FRNA showed that four of the seven poultry workers who were PCR-positive had FRNA titres in the first serum samples. Among the second serum samples, obtained 14 days later, seroconversion was detected in four samples including the person from whom the virus was isolated. One of the workers had a four-fold increase in FRNA titre in the second sample compared with the titre in the first serum sample. Analysis of the third serum samples, obtained 44 days after the first, revealed a decrease in FRNA titres in comparison with the second sample (Table). The maximum observed HIA titre in the second and third serum samples was 1:20 (with the lowest serum dilution in the assay 1:20), which has not been considered previously as a relevant titre for the diagnosis of zoonotic influenza infection. The presence of specific IgG antibodies against influenza A/Astrakhan/3212/2020 was confirmed using the BLI method for five serum samples obtained on the 14th day and for all samples obtained on the 44th day (Supplementary Figure S1).

**Table:** Molecular testing of human serum and nasopharyngeal samples, influenza A(H5N8) on a poultry farm, Astrakhan, Russia, December 2020 (n = 7)

Case	HI reciprocal titres			FRNA reciprocal titres			PCR results		Partial sequencing results
	First serum	Second serum 14 days later	Third serum 44 days later	First serum	Second serum 14 days later	Third serum 44 days later	Influenza A, Cq^a^	Influenza A(H5), Cq^a^	
1	< 20	< 20	< 20	20	20	< 20	21.81	27.19	H5N8
2	< 20	< 20	< 20	20	80	< 20	27.44	> 35	H5N8
3	< 20	< 20	< 20	< 20	< 20	< 20	27.59	25.01	H5N8
4	< 20	< 20	< 20	< 20	< 20	< 20	24.31	21.33	H5N8
5^b^	< 20	< 20	20	< 20	20	20	23.04	23.34	H5N8
6	< 20	20	< 20	20	40	< 20	28.54	> 35	H5N8
7	< 20	20	20	20	40	20	27.79	26.30	H5N8

Cq: quantification cycle; HI: haemagglutinin inhibition; FRNA: focus reduction neutralisation assay.

^a^ Excluding 10 dark cycles.

^b^ Influenza A(H5N8) virus was isolated from this case.

## Virus characterisation

Whole genome sequencing was performed for the influenza A/Astrakhan/3212/2020 strain and for five avian influenza A(H5N8) ECE isolates (A/chicken/Astrakhan/321-01/2020, A/chicken/Astrakhan/321-05/2020, A/chicken/Astrakhan/321-06/2020, A/chicken/Astrakhan/321-09/2020, A/chicken/Astrakhan/321-10/2020) and tissue material from two birds as described previously [2]. The sequence data were deposited in the Global Initiative on Sharing All Influenza Data (GISAID) EpiFlu database (EPI_ISL_1038924, EPI_ISL_1039231, EPI_ISL_1039234, EPI_ISL_1039236, EPI_ISL_1039238, EPI_ISL_1039240) [3]. Phylogenetic analysis indicated that all studied viruses belonged to clade 2.3.4.4b (Figure). An eight-segment constellation clustered the A/Astrakhan/3212/2020 virus with a group of reassortants that have circulated in a wide Eurasian region since July 2020 [4].

**Figure:**
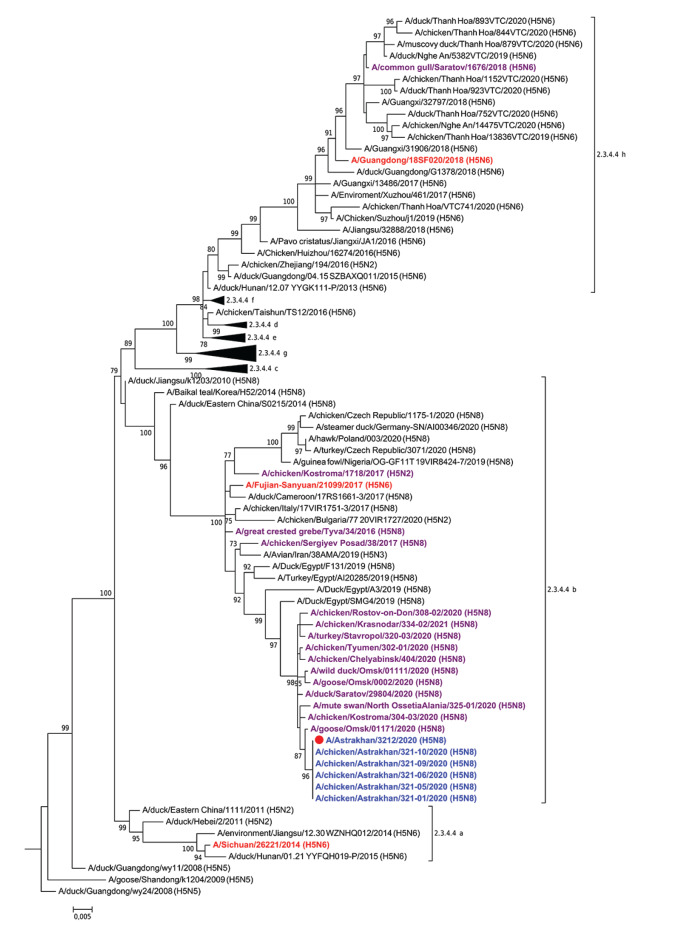
Phylogenetic relationships of the HA gene of influenza A(H5Nx) clade 2.3.4.4 viruses, influenza A(H5N8) on a poultry farm, Astrakhan, Russia, December 2020

Newly characterised viruses isolated in the Astrakhan region are indicated in blue. Viruses previously isolated in Russia are indicated in purple. Candidate vaccine viruses are indicated in red. Haemagglutinin sequences from reference strains used in the analysis were obtained from the EpiFlu database of GISAID (Supplementary Table S2). Bootstrap support values (500 replicates) are indicated at each node. The tree was constructed using MEGA 6.06 software (http://www.megasoftware.net/).

All five viruses isolated from birds and one human isolate showed high genetic similarity of the HA gene with that of other clade 2.3.4.4b viruses detected in poultry and wild birds from 2016 to 2021 in Russia.

Comparison of genome sequences revealed that the MDCK human isolate A/Astrakhan/3212/2020 HA and NA genes were identical to the ECE avian isolate A/chicken/Astrakhan/321–06/2020 at amino acid level. The human isolate and one avian isolate had an amino acid substitution S28N in NA that was not found in the other four avian isolates sequenced. Partial sequences of the HA gene in the clinical specimens from seven cases (Table), obtained for the purpose of subtyping, were identical to the A/Astrakhan/3212/2020 strain and to our five avian influenza A(H5N8) isolates. Partial sequencing of the NA gene from the seven clinical samples showed that all samples had the 28S amino acid (Supplementary Table S1). Both virus variants, with 28N and 28S in NA, were present in the avian population. The absence of 28N in NA sequences of clinical specimens could be due to bias that may have been introduced by using nested PCR [5]. The amino acid substitution S28N has been found only in 0.4% (7/1,639) of influenza A(H5N8) cases worldwide (GISAID, accessed on 30 March 2021). The amino acid 28N (N8 numbering) in NA is also present in several candidate vaccine viruses (CVV) that are known to infect humans: A/Fujian-Sanyuan/21099/2017(H5N6), A/Sichuan/26221/2014(H5N6), A/Hubei/1/2010(H5N1) and A/Anhui/1/2013(H7N9). Currently, no information on the phenotypic significance of the mutation is available. The strain A/Astrakhan/3212/2020, compared with our five sequenced avian isolates, had an additional amino acid substitution in the polymerase acidic protein (PA) gene A598T (single nucleotide polymorphism analysis showed nearly equal proportions of 598T and 598A in the human isolate).

Strain A/Astrakhan/3212/2020 had the polybasic proteolytic cleavage site (PLREKRRKR/G) in HA. Comparison of A/Astrakhan/3212/2020 with the closest antigenic reference strain of clade 2.3.4.4b A/Fujian-Sanyuan/21099/2017 (CVV) showed that among the three amino acid substitutions detected in HA1, one, T140A in antigenic site A, may be associated with antigenic drift. Our strain A/Astrakhan/3212/2020 and CVV A/Fujian-Sanyuan/21099/2017 had the same receptor-binding site (RBS) markers. Both viruses had a QS(R)G motif at the RBS (nt 222–224), which is associated with an avian-like α2,3-sialic acid (SA) receptor-binding preference (H5 numbering) [6]. The amino acid markers associated with increased α2–6 sialoside-binding specificity in the HA gene included 123P, 133A and 156A. 

Genotypic analysis showed that A/Astrakhan/3212/2020 did not have mutations associated with reduced susceptibility to NA inhibitors, adamantanes or baloxavir marboxil [7]. Phenotypic analysis of the human and avian isolates showed normal susceptibility to oseltamivir and zanamivir.

## Ethical statement

The study of clinical material was approved by the Ethics Committee IRB 00001360 affiliated with the Federal Budgetary Research Institution State Research Centre of Virology and Biotechnology ‘Vector’ (No.1-E/21/03/20 Protocol, March 2020).

## Discussion

The wide spread of the HPAI A(H5N8) virus of clade 2.3.4.4 has been observed in Asia, Russia, Europe and North America in recent years [2,8,9]. These epizooties have resulted in several economically costly poultry outbreaks and continue to pose a risk to agriculture and humans. Influenza viruses of the H5N6 subtype of clade 2.3.4.4 have been known to infect humans since 2014, and several cases have resulted in fatal outcomes [10,11]. Because of the potential risk of a pandemic, constant surveillance of the clade 2.3.4.4 A(H5) is considered essential [12]. 

Here, we report human infections with influenza A(H5N8) clade 2.3.4.4b virus in Russia during a poultry outbreak. The reported cases were asymptomatic. The absence of seroconversion observed in two of the five cases could possibly be explained by either insufficient sensitivity of the used methods or may be an occurrence of nasal carriage or local virus replication. The HA gene of the A/Astrakhan/3212/2020 virus isolated from one human case was genetically similar to the HA gene of other H5N8 viruses recently detected in circulation and to the CVV A/Fujian-Sanyuan/21099/2017. As reported in the WHO review on zoonotic influenza in March 2021, these viruses reacted well with post-infection ferret antisera raised against the influenza A(H5N6) A/Fujian-Sanyuan/21099/2017 CVV [10]. Genetic analysis did not show significant differences in RBS from other A(H5) viruses of clade 2.3.4.4b. Most of the experimentally studied clade 2.3.4.4 viruses with the same mutations exhibit dual receptor specificity [12,13]. Our analysis suggests that A/Astrakhan/3212/2020 may have an affinity for both avian- and human-type receptors. Human-to-human transmission of clade 2.3.4.4 viruses has not been reported to date [12]. It is important to monitor the occurrence of additional RBS adaptation of the viruses to human-type receptors in order to evaluate their potential for increased avian-to-human and possibly human-to-human transmissibility. HPAI A(H5N8) viruses can possibly acquire higher virulence and increased transmission properties through mutations and reassortment.

## Conclusion

This case of human infection with influenza A(H5N8) of clade 2.3.4.4 and other reports of sporadic human infections with viruses of this clade indicate a possible risk of an increase in the ability of these viruses to cross the species barrier. The continued spread of HPAI A(H5Nx) viruses, poultry outbreaks, human exposure and infections result in a high demand for enhanced surveillance and increased protection measures for people at risk and strategic measures to protect the human population from possible HPAI pandemics.

## Supplementary Data

219000 Supplement
