# Supplementary material for: Isolation of clade 2.3.4.4b A(H5N8), a highly pathogenic avian influenza virus, from a worker during an outbreak on a poultry farm, Russia, December 2020
Source: Euro Surveill. 2021 Jun 17;26(24):2100439. doi: 10.2807/1560-7917.ES.2021.26.24.2100439 (PMC8212591; doi:10.2807/1560-7917.ES.2021.26.24.2100439)
Supplement: Supplement [file 21-00439_SUSLOPAROV_Supplement.pdf]

This supplementary material is hosted by *Eurosurveillance* as supporting information alongside the article [Isolation of clade 2.3.4.4b A(H5N8), a highly pathogenic avian influenza virus from a human in Russia in 2020], on behalf of the authors, who remain responsible for the accuracy and appropriateness of the content. The same standards for ethics, copyright, attributions and permissions as for the article apply. Supplements are not edited by *Eurosurveillance* and the journal is not responsible for the maintenance of any links or email addresses provided therein.

Supplementary Table S1. Primers designed for nested PCR. The primers were used for subtyping human swab samples for H5N8 (case numbers 1-7). Amplicons were sequenced using NGS and Sanger sequencing. Sequencing results are provided below.

**N8 NA amplicon 1**	Nucleotide position
AST_F1: 5′-CCA AAT CAG AAA ATA GCG ACC-3′	27-47
AST_R1: 5′-CAA AGC CCT TGA CAT CAC-3′	289-306
(Nested PCR) AST_nF2: 5′-GAC CAT TGG CTC CAT CTC-3′	44-61
(Nested PCR) AST_nR2: 5′-CCT CGT TCC AAT GCG G-3′	243-258
**N8 NA amplicon 2**	
AST_F3: 5′-GGC GTA AAA GGT TTC GGG-3′	1053-1070
AST_R3: 5′-CTC CCA GAC AAT TCT ACT GG-3′	1245-1264
(Nested PCR) AST_nF4: 5′-GGA ACT GAT GTG TGG ATG GG-3′	1080-1099
(Nested PCR) AST_nR4: 5′-AAT TCT ACT GGT AAA GTG AAA GAC CC-3′	1230-1255
**H5 HA amplicon 1**	
AST_F5: 5′-CGG AAT GGT CCT ACA TAG TGG-3′	297-317
AST_R5: 5′-CAC AGT ATC AAG AGA TCT TCC CG-3′	581-603
(Nested PCR) AST_nF6: 5′-CAG CTA ATG ACC TCT GCT ACC-3′	330-350
(Nested PCR) AST_nR6: 5′-GTA TTA TTG TAG CTT ATC TTT ATC GTT GGG-3′	547-576
**H5 HA 2 amplicon 2**	
AST_F7: 5′-CGC TGC AGA CAA AGA ATC C-3′	1165-1183
AST_R7: 5′-CCA TGA GAA CTA GAA GTT CAG C-3′	1349-1370
(Nested PCR) AST_nF8: 5′-GCA ATA GAT GGA GTT ACC AAT AAG G-3′	1193-1217
(Nested PCR) AST_nR8: 5′-TCC AGA CAT CTA GGA ATC CG-3′	1321-1340

Fragmentary sequencing results for human cases of H5N8 (case number 1-7):

N8 NA amplicon 1
> Case number 1_Sanger H5N8 NA8 amplicon 1
CATGCCTTGAGCATCATATTAATGGTGTTAGCCCTGGGGAAAAGTGAAAACAATGG
> Case number 2_Sanger H5N8 NA8 amplicon 1
CATGCCTTGAGCATCATATTAATGGTGTTAGCCCTGGGGAAAAGTGAAAACAATGG
> Case number 3_NGS H5N8 NA8 amplicon 1
ATTGGGACTAGTTGTATTCAATGTTCTACTGCATGCCTTGAGCATCATATTAATGGTGTTAGCCCTGGGGAAAAGTGAAAACAATGGAATCTGCAAGGGAACTATAATAAGGGAATATAATGAAACAGTTAGGATAGAGAAAGTGACCCAGTGGTACAACACTAGTGTAGTCGAATATGTA
> Case number 4_NGS H5N8 NA8 amplicon 1
ATTGGGACTAGTTGTATTCAATGTTCTACTGCATGCCTTGAGCATCATATTAATGGTGTTAGCCCTGGGGAAAAGTGAAAACAATGGAATCTGCAAGGGAACTATAATAAGGGAATATAATGAAACAGTTAGGATAGAGAAAGTGACCCAGTGGTACAACACTAGTGTAGTCGAATATGTA
> Case number 5_NGS H5N8 NA8 amplicon 1
ATTGGGACTAGTTGTATTCAATGTTCTACTGCATGCCTTGAGCATCATATTAATGGTGTTAGCCCTGGGGAAAAGTGAAAACAATGGAATCTGCAAGGGAACTATAATAAGGGAATATAATGAAACAGTTAGGATAGAGAAAGTGACCCAGTGGTACAACACTAGTGTAGTCGAATATGTA
> Case number 6_NGS H5N8 NA8 amplicon 1
ATTGGGACTAGTTGTATTCAATGTTCTACTGCATGCCTTGAGCATCATATTAATGGTGTTAGCCCTGGGGAAAAGTGAAAACAATAGAATCTGCAAGGGAACTATAGTAAGGGAATATAATGAAACAGTTAGGATAGAGAAAGTGACCCAGTGGTACAACACTAGTGTAGTCGAATATGTA

> Case number 7_NGS H5N8 NA8 amplicon 1
ATTGGGACTAGTTGTATTCAATGTTCTACTGCATGCCTTGAGCATCATATTAATGGTGTTAGCCCTGGGGAAAAGT
GAAAACAATGGAATCTGCAAGGGAACTATAATAAGGGAATATAATGAAACAGTTAGGATAGAGAAAGTGACCCAGT
GGTACAACACTAGTGTAGTCGAATATGTA
> Case number 7_NGS H5N8 NA8 amplicon 1_minor virus variant
ATTGGGACTAGTTGTATTCAATGTTCTACTGCATGCCTTGAGCATCATATTAATGGTGTTAGCCCTGGGGAAAAGT
GAAAACAATAGAATCTGCAAGGGAACTATAGTAAGGGAATATAATGAAACAGTTAGGATAGAGAAAGTGACCCAGT
GGTACAACACTAGTGTAGTCGAATATGTA

N8 NA amplicon 2
> Case number 3_NGS H5N8 NA8 amplicon 2
GCGGACAATTAGTCGAACTTCCAGGTCAGGGTTTGAAATAATAAGGATAAAGAATGGTTGGACGCAGACAAGCAAA
GAACAGATTAGAAGGCAAGTGGTTGTTGATAATTTGAATTGGTCGGGATACAGT
> Case number 3_NGS H5N8 NA8 amplicon 2_minor virus variant
GCGGACAATTAGTCGAACCTCCAGGTCAGGGTTTGAAATAATAAGGATAAAGAATGGTTGGACGCAGACAAGCAA
AGAACAGATTAGAAGGCAGGTGGTTGTTGGTAATTTGAATTGGTCGGGATACAGT
> Case number 4_NGS H5N8 NA8 amplicon 2
GCGGACAATTAGTCGAACTTCCAGGTCAGGGTTTGAAATAATAAGGATAAAGAATGGTTGGACGCAGACAAGCAAA
GAACAGATTAGAAGGCAAGTGGTTGTTGATAATTTGAATTGGTCGGGATACAGT
> Case number 5_NGS H5N8 NA8 amplicon 2
GCGGACAATTAGTCGAACTTCCAGGTCAGGGTTTGAAATAATAAGGATAAAGAATGGTTGGACGCAGACAAGCAAA
GAACAGATTAGAAGGCAAGTGGTTGTTGATAATTTGAATTGGTCGGGATACAGT
> Case number 5_NGS H5N8 NA8 amplicon 2_minor virus variant
GCGGACAATTAGTCGAACCTCCAGGTCAGGGTTTGAAATAATAAGGATAAAGAATGGTTGGACGCAGACAAGCAA
AGAACAGATTAGAAGGCAGGTGGTTGTTGGTAATTTGAATTGGTCGGGATACAGT
> Case number 7_NGS H5N8 NA8 amplicon 2
GCGGACAATTAGTCGAACCTCCAGGTCAGGGTTTGAAATAATAAGGATAAAGAATGGTTGGACGCAGACAAGCAA
AGAACAGATTAGAAGGCAGGTGGTTGTTGGTAATTTGAATTGGTCGGGATACAGT
> Case number 7_NGS H5N8 NA8 amplicon 2_minor virus variant
GCGGACAATTAGTCGAACTTCCAGGTCAGGGTTTGAAATAATAAGGATAAAGAATGGTTGGACGCAGACAAGCAAA
GAACAGATTAGAAGGCAAGTGGTTGTTGATAATTTGAATTGGTCGGGATACAGT

H5 HA amplicon 1
> Case number 2_NGS H5N8 HA5 amplicon 1
CAGGGAGCCTCAATGACTATGAAGAACTGAAACACCTGTTGAGCAGAATAAATCATTTTGAGAAGATTCTGATTAT
CCCCAAGAGTTCCTGGCCAAACCATGAAACATCACTAGGGGTGAGCGCAGCTTGTCCATACCAGGGAGCGCCCTCC
TTTTTCAGAAATGTGGTGTGGCTTATCAAAAAGAACGATGCATA
> Case number 3_NGS H5N8 HA5 amplicon 1
CAGGGAGCCTCAATGACTATGAAGAACTGAAACACCTGTTGAGCAGAATAAATCATTTTGAGAAGATTCTGATTAT
CCCCAAGAGTTCCTGGCCAAACCATGAAACATCACTAGGGGTGAGCGCAGCTTGTCCATACCAGGGAGCGCCCTCC
TTTTTCAGAAATGTGGTGTGGCTTATCAAAAAGAACGATGCATA
> Case number 4_NGS H5N8 HA5 amplicon 1
CAGGGAGCCTCAATGACTATGAAGAACTGAAACACCTGTTGAGCAGAATAAATCATTTTGAGAAGATTCTGATTAT
CCCCAAGAGTTCCTGGCCAAACCATGAAACATCACTAGGGGTGAGCGCAGCTTGTCCATACCAGGGAGCGCCCTCC
TTTTTCAGAAATGTGGTGTGGCTTATCAAAAAGAACGATGCATA
> Case number 5_NGS H5N8 HA5 amplicon 1
CAGGGAGCCTCAATGACTATGAAGAACTGAAACACCTGTTGAGCAGAATAAATCATTTTGAGAAGATTCTGATTAT
CCCCAAGAGTTCCTGGCCAAACCATGAAACATCACTAGGGGTGAGCGCAGCTTGTCCATACCAGGGAGCGCCCTCC
TTTTTCAGAAATGTGGTGTGGCTTATCAAAAAGAACGATGCATA
> Case number 7_NGS H5N8 HA5 amplicon 1
CAGGGAGCCTCAATGACTATGAAGAACTGAAACACCTGTTGAGCAGAATAAATCATTTTGAGAAGATTCTGATTAT
CCCCAAGAGTTCCTGGCCAAACCATGAAACATCACTAGGGGTGAGCGCAGCTTGTCCATACCAGGGAGCGCCCTCC
TTTTTCAGAAATGTGGTGTGGCTTATCAAAAAGAACGATGCATA
> Case number 7_NGS H5N8 HA5 amplicon 1_minor virus variant
CAGGGAGCCTCAATGACTATGAAGAACTGAAACACCTGTTGAGCAGAATAAATCATTTTGAGAAGATTCTGATCAT
CCCCAAGAGTTCCTGGCCAAATCATGAAACATCACTAGGGGTGAGCGCAGCTTGTCCATACCAGGGAGCGCCCTCC
TTTTTCAGAAATGTGGTGTGGCTTATCAAAAAGAACGATGCATA

H5 HA amplicon 2
> Case number 1_NGS H5N8 HA5 amplicon 2

GTCAACTCAATCATTGACAAAATGAACACTCAATTTGAGGCAGTTGGAAGGGAGTTTAATAACTTAGAAAGGAGGA
TAGAGAATTTGAACAAGAAAATGGAAGA
> Case number 2_NGS H5N8 HA5 amplicon 2
GTCAACTCAATCATTGACAAAATGAACACTCAATTTGAGGCAGTTGGAAGGGAGTTTAATAACTTAGAAAGGAGGA
TAGAGAATTTGAACAAGAAAATGGAAGA
> Case number 3_NGS H5N8 HA5 amplicon 2
GTCAACTCAATCATTGACAAAATGAACACTCAATTTGAGGCAGTTGGAAGGGAGTTTAATAACTTAGAAAGGAGGA
TAGAGAATTTGAACAAGAAAATGGAAGA
> Case number 4_NGS H5N8 HA5 amplicon 2
GTCAACTCAATCATTGACAAAATGAACACTCAATTTGAGGCAGTTGGAAGGGAGTTTAATAACTTAGAAAGGAGGA
TAGAGAATTTGAACAAGAAAATGGAAGA
> Case number 5_NGS H5N8 HA5 amplicon 2
GTCAACTCAATCATTGACAAAATGAACACTCAATTTGAGGCAGTTGGAAGGGAGTTTAATAACTTAGAAAGGAGGA
TAGAGAATTTGAACAAGAAAATGGAAGA
> Case number 6_NGS H5N8 HA5 amplicon 2
GTCAACTCAATCATTGACAAAATGAACACTCAATTTGAGGCAGTTGGAAGGGAGTTTAATAACTTAGAAAGGAGGA
TAGAGAATTTGAACAAGAAAATGGAAGA
> Case number 7_NGS H5N8 HA5 amplicon 2
GTCAACTCAATCATTGACAAAATGAACACTCAATTTGAGGCAGTTGGAAGGGAGTTTAATAACTTAGAAAGGAGGA
TAGAGAATTTGAACAAGAAAATGGAAGA

Commentary to the sequencing results:

H5 HA amplicon 2 sequences of original samples of all seven cases (numbers 1-7) were identical to A/Astrakhan/3212/2020.

H5 HA amplicon 1 was sequenced for original samples of five cases. Sequences of major virus variants were identical to A/Astrakhan/3212/202. One sample of case number 7 had additional minor virus variant with synonymous nucleotide substitutions T424C and C448T. These SNPs were detected in the original material of A/chicken/Astrakhan/321-10/2020 with proportion >5%.

N8 NA amplicon 1 was sequenced for original samples of all seven viruses. All seven sequences, had one nucleotide substitution A103G (protein N28S) in comparison to A/Astrakhan/3212/2020 (same substitution was found in four of the five sequenced avian Astrakhan H5N8 isolates of the study). In addition, sequences of the original samples of case number 6 (major) and case number 7 (minor) had two nucleotide substitutions G147A (protein G43R), A168G (protein I50V). The SNPs G147A and A168G were detected in the original material of A/chicken/Astrakhan/321-10/2020 with proportion >5%.

N8 NA amplicon 2 was sequenced for original samples of four cases. The sequences were identical to A/Astrakhan/3212/2020, except sequences of minor virus variants of original samples of case numbers 3, 5 and major variant of case number 7, which had three nucleotide substitutions T1118C, A1193G (synonymous) and A1204G (protein D395G). These SNPs were detected in the original material of A/chicken/Astrakhan/321-10/2020 with proportion >5%.

Supplementary Figure S1. Bio-layer interferometry (BLI) curves for binding of specific IgG antibodies to the A/Astrakhan/3212/2020 in the serum samples on the Octet RED96e system. A: Serum binding samples obtained on the 14th day; **B**: samples obtained on the 44th day.

Commentary:
1 – 7: serum samples; 8 – negative control.
Vitus purification: Allantoic fluid was clarified by combination of centrifugation, filtration and size-exclusion chromatography.
Virus Binding Assay: Virus binding was measured using an Octet RED96e system (Pall ForteBio, CA). Serum diluted 1:10 in sample buffer were loaded onto Protein A biosensors (Pall ForteBio) for 1000 s to maximum saturation. Binding of A/Astrakhan/3212/2020 strain at 64 hemagglutinating units was measured for 25-50 min. All experiments were carried out in kinetic buffer.
Sera were bound to eight biosensors, and after removing excess serum, the biosensors were incubated with A/Astrakhan/3212/2020. Each curve is a separate dilution of serum. Binding results in a wavelength shift measured in nanometers.

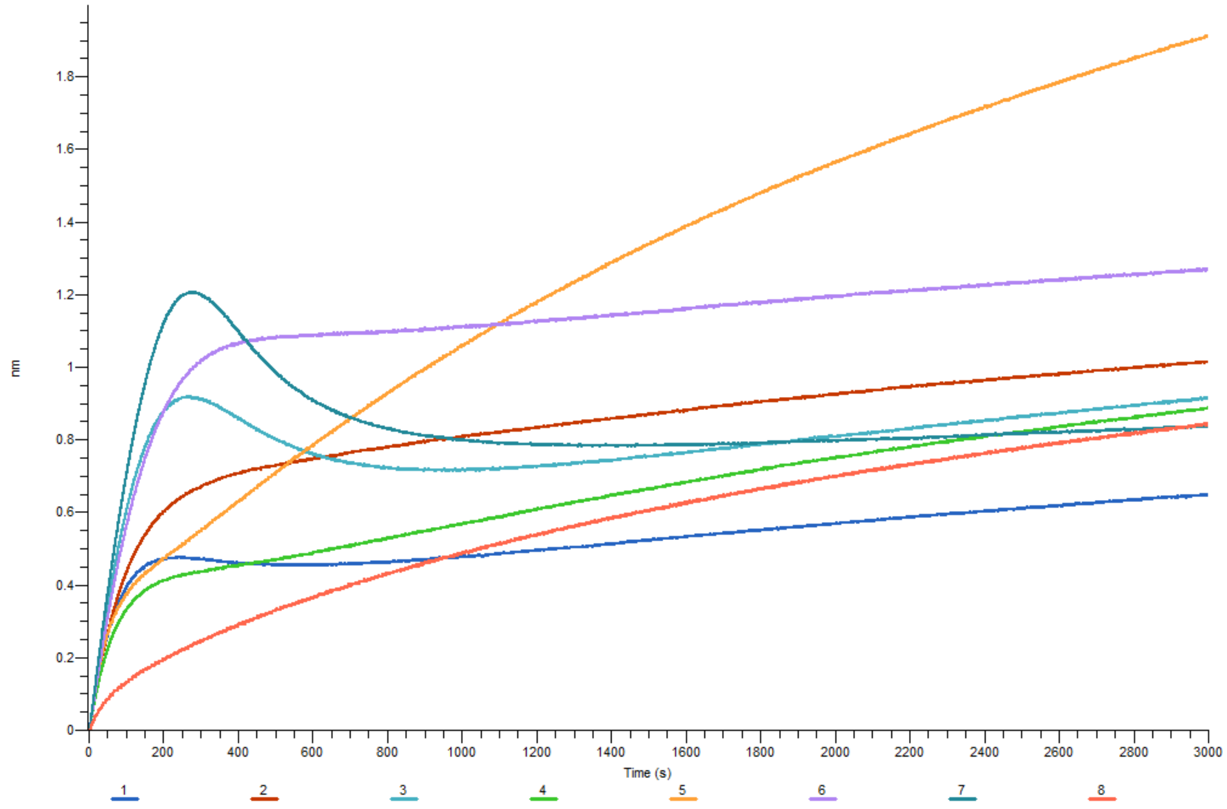

nm
Time (s)
0
0.2
0.4
0.6
0.8
1
1.2
1.4
1.6
1.8
0
200
400
600
800
1000
1200
1400
1600
1800
2000
2200
2400
2600
2800
3000
1
2
3
4
5
6
7
8

A

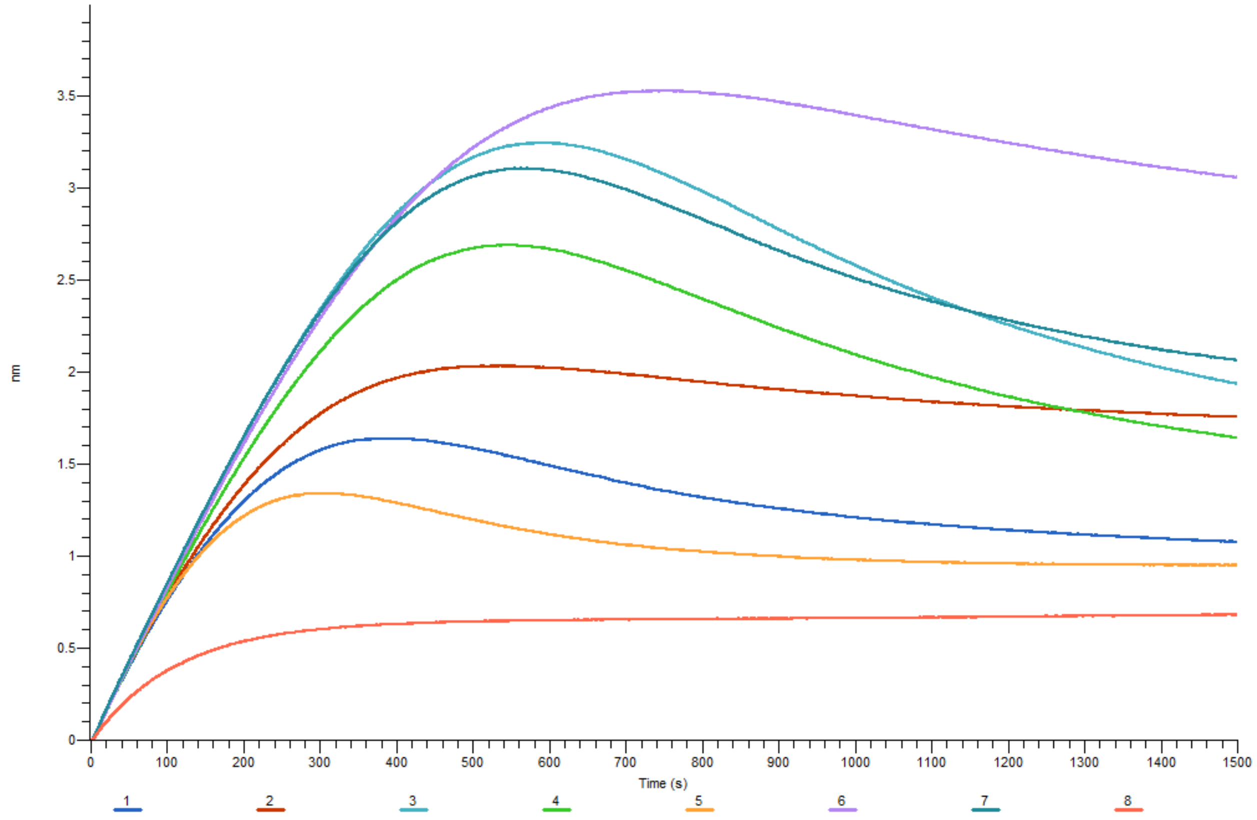

nm
Time (s)
0
0.5
1
1.5
2
2.5
3
3.5
0
100
200
300
400
500
600
700
800
900
1000
1100
1200
1300
1400
1500
1
2
3
4
5
6
7
8

B

Supplementary Table S2. Origin of haemagglutinin sequences of influenza A(H5) isolates used for the phylogenetic analysis.

Segment ID	Segment	Country	Collection date	Isolate name	Originating Lab	Submitting Lab	Authors
EPI1355418	HA	Russian Federation	2018-Oct-01	A/common gull/Saratov/1676/2018	State Research Center of Virology and Biotechnology (VECTOR)	State Research Center of Virology and Biotechnology (VECTOR)	Natalia,Goncharova; Ivan,Susloparov; Natalia,Kolosova; Alexey,Danilenko; Juliya,Bulanovich; Vasiliy,Marchenko; Alexander,Ryzhikov
EPI1848718	HA	Russian Federation	2020-Dec-31	A/mute swan/North Ossetia-Alania/325-01/2020	State Research Center of Virology and Biotechnology (VECTOR)	State Research Center of Virology and Biotechnology (VECTOR)	Natalia,Goncharova; Ivan,Susloparov; Natalia,Kolosova; Alexey,Danilenko; Juliya,Bulanovich; Vasiliy,Marchenko; Alexander,Ryzhikov
EPI1846961	HA	Russian Federation	2020-Dec-12	A/Astrakhan/3212/2020	Center of Hygiene and Epidemiology in Astrakhan Region	State Research Center of Virology and Biotechnology (VECTOR)	Pyankova, O; Susloparov, I; Marchenko, V; Ryzhikov, A
EPI961459	HA	Russian Federation	2017-Mar-02	A/chicken/Sergiyev Posad/38/2017		State Research Center of Virology and Biotechnology (VECTOR)	Ivan,Susloparov; Natalya,Goncharova; Natalya,Kolosova; Vasiliy,Marchenko; Alexander,Ryzhikov
EPI1813249	HA	Russian Federation	2020-Aug-17	A/wild duck/Omsk/01111/2020	State Research Center of Virology and Biotechnology (VECTOR)	State Research Center of Virology and Biotechnology (VECTOR)	Natalia,Goncharova; Ivan,Susloparov; Natalia,Kolosova; Alexey,Danilenko; Juliya,Bulanovich; Vasiliy,Marchenko; Alexander,Ryzhikov
EPI1814473	HA	Vietnam	2020-Feb-20	A/muscovy duck/Thanh Hoa/879VTC/2020	State Research Center of Virology and Biotechnology (VECTOR)	State Research Center of Virology and Biotechnology (VECTOR)	
EPI1848790	HA	Russian Federation	2021-Jan-05	A/chicken/Krasnodar/334-02/2021	State Research Center of Virology and Biotechnology (VECTOR)	State Research Center of Virology and Biotechnology (VECTOR)	Natalia,Goncharova; Ivan,Susloparov; Natalia,Kolosova; Alexey,Danilenko; Juliya,Bulanovich; Vasiliy,Marchenko; Alexander,Ryzhikov

EPI1848670	HA	Russian Federation	2020-Oct-25	A/chicken/Rostov-on-Don/308-02/2020	State Research Center of Virology and Biotechnology (VECTOR)	State Research Center of Virology and Biotechnology (VECTOR)	Natalia,Goncharova; Ivan,Susloparov; Natalia,Kolosova; Alexey,Danilenko; Juliya,Bulanovich; Vasiliy,Marchenko; Alexander,Ryzhikov
EPI1848630	HA	Russian Federation	2020-Oct-17	A/chicken/Kostroma/304-03/2020	State Research Center of Virology and Biotechnology (VECTOR)	State Research Center of Virology and Biotechnology (VECTOR)	Natalia,Goncharova; Ivan,Susloparov; Natalia,Kolosova; Alexey,Danilenko; Juliya,Bulanovich; Vasiliy,Marchenko; Alexander,Ryzhikov
EPI1848606	HA	Russian Federation	2020-Sep-26	A/chicken/Tyumen/302-01/2020	State Research Center of Virology and Biotechnology (VECTOR)	State Research Center of Virology and Biotechnology (VECTOR)	Natalia,Goncharova; Ivan,Susloparov; Natalia,Kolosova; Alexey,Danilenko; Juliya,Bulanovich; Vasiliy,Marchenko; Alexander,Ryzhikov
EPI1847025	HA	Russian Federation	2020-Dec-12	A/chicken/Astrakhan/321-10/2020	State Research Center of Virology and Biotechnology (VECTOR)	State Research Center of Virology and Biotechnology (VECTOR)	Natalia,Goncharova; Ivan,Susloparov; Natalia,Kolosova; Alexey,Danilenko; Juliya,Bulanovich; Vasiliy,Marchenko; Alexander,Ryzhikov
EPI1847009	HA	Russian Federation	2020-Dec-12	A/chicken/Astrakhan/321-09/2020	State Research Center of Virology and Biotechnology (VECTOR)	State Research Center of Virology and Biotechnology (VECTOR)	Natalia,Goncharova; Ivan,Susloparov; Natalia,Kolosova; Alexey,Danilenko; Juliya,Bulanovich; Vasiliy,Marchenko; Alexander,Ryzhikov
EPI1847001	HA	Russian Federation	2020-Dec-12	A/chicken/Astrakhan/321-06/2020	State Research Center of Virology and Biotechnology (VECTOR)	State Research Center of Virology and Biotechnology (VECTOR)	Natalia,Goncharova; Ivan,Susloparov; Natalia,Kolosova; Alexey,Danilenko; Juliya,Bulanovich; Vasiliy,Marchenko; Alexander,Ryzhikov
EPI1846985	HA	Russian Federation	2020-Dec-12	A/chicken/Astrakhan/321-05/2020	State Research Center of Virology and Biotechnology (VECTOR)	State Research Center of Virology and Biotechnology (VECTOR)	

EPI1846969	HA	Russian Federation	2020-Dec-12	A/chicken/Astrakhan/321-01/2020	State Research Center of Virology and Biotechnology (VECTOR)	State Research Center of Virology and Biotechnology (VECTOR)	
EPI1814585	HA	Vietnam	2019-Dec-27	A/chicken/Nghe An/14475VTC/2020	State Research Center of Virology and Biotechnology (VECTOR)	State Research Center of Virology and Biotechnology (VECTOR)	
EPI1814545	HA	Vietnam	2020-Mar-06	A/chicken/Thanh Hoa/1152VTC/2020	State Research Center of Virology and Biotechnology (VECTOR)	State Research Center of Virology and Biotechnology (VECTOR)	
EPI1814465	HA	Vietnam	2020-Feb-18	A/chicken/Thanh Hoa/844VTC/2020	State Research Center of Virology and Biotechnology (VECTOR)	State Research Center of Virology and Biotechnology (VECTOR)	
EPI1814433	HA	Vietnam	2020-Feb-13	A/chicken/Thanh Hoa/VTC741/2020	State Research Center of Virology and Biotechnology (VECTOR)	State Research Center of Virology and Biotechnology (VECTOR)	
EPI1813433	HA	Russian Federation	2020-Aug-06	A/chicken/Chelyabinsk/404/2020	State Research Center of Virology and Biotechnology (VECTOR)	State Research Center of Virology and Biotechnology (VECTOR)	Natalia,Goncharova; Ivan,Susloparov; Natalia,Kolosova; Alexey,Danilenko; Juliya,Bulanovich; Vasiliy,Marchenko; Alexander,Ryzhikov
EPI1665392	HA	Vietnam	2019-Jul-24	A/chicken/Thanh Hoa/13836VTC/2019	State Research Center of Virology and Biotechnology (VECTOR)	State Research Center of Virology and Biotechnology (VECTOR)	
EPI1158108	HA	Russian Federation	2017-Dec-22	A/chicken/Kostroma/1718/2017		State Research Center of Virology and Biotechnology (VECTOR)	Ivan,Susloparov; Natalya,Goncharova; Natalya,Kolosova; Vasiliy,Marchenko; Alexander,Ryzhikov
EPI1813201	HA	Russian Federation	2020-Aug-17	A/goose/Omsk/01171/2020	State Research Center of Virology and Biotechnology (VECTOR)	State Research Center of Virology and Biotechnology (VECTOR)	Natalia,Goncharova; Ivan,Susloparov; Natalia,Kolosova; Alexey,Danilenko; Juliya,Bulanovich; Vasiliy,Marchenko; Alexander,Ryzhikov
EPI1813121	HA	Russian Federation	2020-Aug-13	A/goose/Omsk/0002/2020	State Research Center of Virology and Biotechnology (VECTOR)	State Research Center of Virology and Biotechnology (VECTOR)	Natalia,Goncharova; Ivan,Susloparov; Natalia,Kolosova; Alexey,Danilenko; Juliya,Bulanovich; Vasiliy,Marchenko; Alexander,Ryzhikov
EPI1814497	HA	Vietnam	2020-Feb-22	A/duck/Thanh Hoa/923VTC/2020	State Research Center of Virology and Biotechnology (VECTOR)	State Research Center of Virology and Biotechnology (VECTOR)	

EPI1814489	HA	Vietnam	2020-Feb-21	A/duck/Thanh Hoa/893VTC/2020	State Research Center of Virology and Biotechnology (VECTOR)	State Research Center of Virology and Biotechnology (VECTOR)	
EPI1814441	HA	Vietnam	2020-Feb-13	A/duck/Thanh Hoa/752VTC/2020	State Research Center of Virology and Biotechnology (VECTOR)	State Research Center of Virology and Biotechnology (VECTOR)	
EPI1814265	HA	Russian Federation	2020-Sep-15	A/duck/Saratov/29804/2020	State Research Center of Virology and Biotechnology (VECTOR)	State Research Center of Virology and Biotechnology (VECTOR)	Natalia,Goncharova; Ivan,Susloparov; Natalia,Kolosova; Alexey,Danilenko; Juliya,Bulanovich; Vasiliy,Marchenko; Alexander,Ryzhikov
EPI1665320	HA	Vietnam	2019-Nov-29	A/duck/Nghe An/5382VTC/2019	State Research Center of Virology and Biotechnology (VECTOR)	State Research Center of Virology and Biotechnology (VECTOR)	
EPI1848710	HA	Russian Federation	2020-Dec-11	A/turkey/Stavropol/320-03/2020	State Research Center of Virology and Biotechnology (VECTOR)	State Research Center of Virology and Biotechnology (VECTOR)	Natalia,Goncharova; Ivan,Susloparov; Natalia,Kolosova; Alexey,Danilenko; Juliya,Bulanovich; Vasiliy,Marchenko; Alexander,Ryzhikov
EPI823460	HA	Russian Federation	2016-May-25	A/great crested grebe/Tyva/34/2016	State Research Center of Virology and Biotechnology (VECTOR)	WHO National Influenza Centre Russian Federation	Fadeev,Artem; Komissarov,Andrey; Egorova,Anna; Sintsova,Ksenia; Musaeva,Tamila; Susloparov,Ivan; Marchenko,Vasiliy; Ryzhikov,Aleksandr
EPI493833	HA	China	2008-Jun-06	A/duck/Guangdong/wy24/2008		Import from public-domain	Liu,C.G.; Liu,M.; Liu,F.; Lv,R.; Liu,D.F.; Qu,L.D.; Zhang,Y.
EPI493817	HA	China	2008-Jun-06	A/duck/Guangdong/wy11/2008		Import from public-domain	Liu,C.G.; Liu,M.; Liu,F.; Lv,R.; Liu,D.F.; Qu,L.D.; Zhang,Y.
EPI442001	HA	China	2009-Dec-16	A/goose/Shandong/k1204/2009		Import from public-domain	Zhao,K.; Gu,M.; Zhong,L.; Duan,Z.; Zhang,Y.; Zhu,Y.; Zhao,G.; Zhao,M.; Chen,Z.; Hu,S.; Liu,W.; Liu,X.; Peng,D.
EPI658863	HA	China	2015-Jan-21	A/duck/Hunan/01.21 YYFQH019-P/2015(H5N6)		Institute of Microbiology, Chinese Academy of Sciences	
EPI533583	HA	China	2014-Apr-21	A/Sichuan/26221/2014		WHO Chinese National Influenza Center	

EPI664381	HA	China	2014-Dec-30	A/environment/Jiangsu/12.30 WZNHQ012/2014(Mixed)		Institute of Microbiology, Chinese Academy of Sciences	
EPI431448	HA	China	2011-Dec-01	A/duck/Hebei/2/2011	Institute of Microbiology, Chinese Academy of Sciences	Institute of Microbiology, Chinese Academy of Sciences	Di,Liu; Haigang,Sun; Jinghua,Yan; George F,Gao; Juncai,Ma
EPI398965	HA	China	2011-Jan-01	A/duck/Eastern China/1111/2011		Import from public-domain	Zhao,G.; Gu,X.; Lu,X.; Pan,J.; Duan,Z.; Zhao,K.; Gu,M.; Liu,Q.; He,L.; Chen,J.; Ge,S.; Wang,Y.; Chen,S.; Wang,X.; Peng,D.; Wan,H.; Liu,X.
EPI1634516	HA	Egypt	2019-Mar-28	A/Duck/Egypt/A3/2019		Import from public-domain	Tantawy,A.S.; Sultan,H.; Arafa,A.; Hagag,N.
EPI1634517	HA	Egypt	2019-Jan-06	A/Duck/Egypt/F131/2019		Import from public-domain	Tantawy,A.S.; Sultan,H.; Arafa,A.; Hagag,N.
EPI1634518	HA	Egypt	2019-Jan-23	A/Duck/Egypt/SMG4/2019		Import from public-domain	Tantawy,A.S.; Sultan,H.; Arafa,A.; Hagag,N.
EPI1638797	HA	Egypt	2019-Apr-03	A/Turkey/Egypt/AI20285/2019	Poultry Diseases Department ,Faculty of Veterinary Medicine, Beni-Suef University	Friedrich-Loeffler-Institut	Hassan,KE;El-Kady,MF;Harder,T.
EPI1601906	HA	Iran, Islamic Republic of	2019-Mar-30	A/Avian/Iran/38AMA/2019		Import from public-domain	Shenagari,M.; Jamali,A.; Abdoli,A.
EPI1780073	HA	Bulgaria	2020-Feb-21	A/chicken/Bulgaria/77_20VIR1727/2020		Import from public-domain	Goujgoulova,G.; Slavcheva,I.; Zecchin,B.; Zamperin,G.; Pastori,A.; Schivo,A.; Fusaro,A.; Terregino,C.
EPI961525	HA	Italy	2017-Feb-27	A/chicken/Italy/17VIR1751-3/2017	Istituto Zooprofilattico Sperimentale Delle Venezie	Istituto Zooprofilattico Sperimentale Delle Venezie	Zecchin, B.; Fusaro, A.; Milani, A.; Schivo, A.; Salviato, A.; Zamperin, G.; Marciano, S.; Ormelli, S.; Terregino, C.; Monne, I.
EPI1223854	HA	Cameroon	2017-Jan-01	A/duck/Cameroon/17RS1661-3/2017		Import from public-domain	Wade,A.; Zecchin,B.; Jumbo,S.D.; Fusaro,A.; Taiga,T.; Bianco,A.; Poueme N,R.; Salomoni,A.; Feussom Kameni,J.M.; Zamperin,G.; Kazi,J.P.; Nenkam,R.; Foupouapouognigni,Y.; Abdoulkadiri,S.; Yaya,A.; Monne,I.

EPI1202729	HA	China	2017-Dec-25	A/Fujian-Sanyuan/21099/2017	Fujian Provincial Center for Disease Control and Prevention	WHO Chinese National Influenza Center	
EPI1666962	HA	Nigeria	2019-Jul-05	A/guinea_fowl/Nigeria/OG-GF11T_19VIR8424-7/2019_H5N8	National Veterinary Research Institute	Istituto Zooprofilattico Sperimentale Delle Venezie	Ismaila,Shittu; Tony,Joannis; Bianca,Zecchin; Ambra,Pastori; Alessia,Schivo; Alice,Fusaro
EPI1667598	HA	Czech Republic	2020-Jan-17	A/chicken/Czech Republic/1175-1/2020	State Veterinary Institute Prague	State Veterinary Institute Prague	Nagy,A
EPI1719042	HA	Czech Republic	2020-Feb-17	A/turkey/Czech Republic/3071/2020	State Veterinary Institute Prague	State Veterinary Institute Prague	Nagy,A
EPI1669677	HA	Poland	2020-Jan-06	A/hawk/Poland/003/2020	National Veterinary Research Institut Poland, PIWet-PIB	National Veterinary Research Institut Poland, PIWet-PIB	
EPI1721676	HA	Germany	2020-Mar-26	A/steamer duck/Germany-SN/AI00346/2020	Landesuntersuchungsanstalt für das Gesundheits- und Veterinärwesen Sachsen	Friedrich-Loeffler-Institut	
EPI703602	HA	China	2014-Feb-15	A/duck/Eastern China/S0215/2014		Import from public-domain	Sun,H.; Sun,Y.; Pu,J.; Liu,L.; Li,C.; Xu,G.; Qin,M.; Zhang,Y.; Zhao,H.; Wei,K.; Liu,J.
EPI561495	HA	Korea, Republic of	2014-Jan-20	A/Baikal teal/Korea/H52/2014		Import from public-domain	Jeong,J.; Kang,H.M.; Lee,E.K.; Song,B.M.; Kwon,Y.K.; Kim,H.R.; Choi,K.S.; Kim,J.Y.; Lee,H.J.; Moon,O.K.; Jeong,W.; Choi,J.; Baek,J.H.; Joo,Y.S.; Park,Y.H.; Lee,H.S.; Lee,Y.J.; Lee,Y.-J.; Kang,H.-M.; Lee,E.-K.; Song,B.-M.; Lee,K.-J.; Hong,M.-S.
EPI442017	HA	China	2010-Dec-05	A/duck/Jiangsu/k1203/2010		Import from public-domain	Zhao,K.; Gu,M.; Zhong,L.; Duan,Z.; Zhang,Y.; Zhu,Y.; Zhao,G.; Zhao,M.; Chen,Z.; Hu,S.; Liu,W.; Liu,X.; Peng,D.
EPI895119	HA	China	2016-Feb-19	A/chicken/Taishun/TS12/2016		Import from public-domain	Chen,L.J.; Tian,J.H.; Lin,X.D.; Liao,Y.; Shi,M.; Zhang,Y.Z.
EPI656642	HA	China	2013-Dec-07	A/duck/Hunan/12.07 YYGK111-P/2013(H5N6)		Institute of Microbiology, Chinese Academy of Sciences	
EPI661943	HA	China	2015-Apr-15	A/duck/Guangdong/04.15 SZBAXQ011/2015(H5N6)		Institute of Microbiology, Chinese Academy of Sciences	

EPI1255953	HA	China	2018-Mar-13	A/duck/Guangdong/G1378/2018		China Animal Health and Epidemiology Center (CAHEC)	
EPI1352813	HA	China	2018-Sep-29	A/Guangdong/18SF020/2018	Guangdong Provincial Center for Disease Control and Prevention	WHO Chinese National Influenza Center	Wang,Dayan;Zhou,Shumei;Li,Xiyan;Liu,Jia;Zhang,Ye;Bo,Hong;Shu,Yuelong
EPI942916	HA	China	2016-Jan-31	A/chicken/Zhejiang/194/2016		Import from public-domain	Wu,H.; Lu,R.; Peng,X.; Chen,B.; Cheng,L.; Wu,N.
EPI1060731	HA	China	2016-Mar-01	A/Chicken/Huizhou/16274/2016(H5N6)	South China Agricultural University	South China Agricultural University	
EPI1352805	HA	China	2018-Aug-15	A/Guangxi/31906/2018	Guangxi Center for Disease Prevention and Control	WHO Chinese National Influenza Center	Wang,Dayan;Zhou,Shumei;Li,Xiyan;Liu,Jia;Zhang,Ye;Bo,Hong;Shu,Yuelong
EPI1352829	HA	China	2018-Oct-25	A/Guangxi/32797/2018	Guangxi Center for Disease Prevention and Control	WHO Chinese National Influenza Center	Wang,Dayan;Zhou,Shumei;Li,Xiyan;Liu,Jia;Zhang,Ye;Bo,Hong;Shu,Yuelong
EPI1352861	HA	China	2017-Nov-17	A/Guangxi/13486/2017	Guangxi Center for Disease Prevention and Control	WHO Chinese National Influenza Center	Wang,Dayan;Zhou,Shumei;Li,Xiyan;Liu,Jia;Zhang,Ye;Bo,Hong;Shu,Yuelong
EPI968994	HA	China	2016-Feb-27	A/Pavo cristatus/Jiangxi/JA1/2016		Import from public-domain	Li,M.; Zhao,N.; Luo,J.; Li,Y.; Chen,L.; Ma,J.; Zhao,L.; Yuan,G.; Wang,C.; Wang,Y.; Liu,Y.; He,H.
EPI1352821	HA	China	2018-Nov-06	A/Jiangsu/32888/2018	Jiangsu Provincial Center for Disease Control & Prevention	WHO Chinese National Influenza Center	Wang,Dayan;Zhou,Shumei;Li,Xiyan;Liu,Jia;Zhang,Ye;Bo,Hong;Shu,Yuelong
EPI1426910	HA	China	2019-Feb-12	A/Chicken/Suzhou/j1/2019	Jiangsu Provincial Center for Disease Control and Prevention	Jiangsu Provincial Center for Disease Control & Prevention	Xian, Qi; Huiyan,Yu
EPI1258057	HA	China	2018-Jan-20	A/Enviroment/Xuzhou/461/2017		Jiangsu Provincial Center for Disease Control & Prevention	
